# Dermatology

**Published:** 1902-09

**Authors:** Henry Waldo


### DERMATOLOGY.

**The Relative Inefficiency of Cacodylates as Therapeutic Agents** is the title of a paper communicated by Dr. T. R. Fraser.[1] It appeared to him that if cacodylic compounds are incapable of causing toxic effects when given in quantities greatly superior to the quantities of ordinarily employed arsenical compounds, they must, to a corresponding degree, be pharmacologically inert and therapeutically inefficient. To test this opinion Dr. Fraser administered cacodylate of soda and cacodylate of iron in several cases of chorea and other diseases, and came to the conclusion that the arsenic in cacodylic acid and in its salts is almost inert when regarded either as a therapeutic or as a poisonous substance; but that when certain bases having therapeutic properties, such as iron, are united with cacodylic

[1] *Scottish M. & S. J.*, 1902, x. 385.

acid, these bases are capable of producing their special curative effects.

Dr. Fraser found that the well-known reactions of the ordinary arsenical compounds are not displayed by cacodylates, while the reactions of the bases referred to can readily be produced. He shows that cacodylates, though arsenate compounds, fail to give the characteristic reactions of sodium arsenate, and that in marked contrast with sodium arsenate the cacodylate of soda also fails to produce a deposit of arsenium upon copper when it is subjected to Reinsch's process. On the other hand, cacodylate of iron gives the numerous ordinary reactions of all solutions of ferric compounds. As contrasted with the iron, the arsenic is shown to be so firmly united with other elements in the cacodylic group as to be incapable of disassociation by chemical reagents of much greater activity than those which it could encounter in the living body. Dr. Fraser therefore suggests that when a cacodylate is administered the arsenic remains locked up in it, in so far as its pharmacological energies are concerned, and is therapeutically inert; and the arsenic probably passes through the body without undergoing disassociation from methyl. He goes on to say that clinical observations and chemical analyses agree in showing that when a salt of cacodylic acid is administered to a patient it is absorbed and is eliminated; but with the arsenic which it contains so firmly combined with the other cacodyl constituents that it does not become disassociated, and is therefore incapable of forming any compound in the body that can produce the well-known pharmacological activities of the compounds of arsenic usually employed for therapeutic purposes. He considers that the compounds of cacodylic acid may thus be given in massive doses, and by them arsenic may be introduced into the body in quantities which are large, and even enormous, relatively to the quantities in the commonly used compounds of arsenic; in the latter this element, however, is not so stably fixed as to be incapable of forming new combinations which produce well-known and powerful effects. He thinks cacodylates may be likened to certain of the cyanogen compounds, such as the ferro- and ferri-cyanides, in which cyanogen is also so locked up that enormous quantities may be given without causing any cyanogen action, or any manifestation of the deadly energy of hydrocyanic acid and the majority of the cyanides.

Dr. Fraser concludes his paper by regarding cacodylates as useless therapeutic agents in so far as they are compounds of arsenic; and besides this he considers they are inconvenient substances to administer to patients, as when given by the mouth they are liable to induce disorders of the alimentary canal, such as nausea, vomiting and diarrhœa. Given by either subcutaneous injection or by the mouth, the highly obnoxious garlic-like odour of cacodylic oxide is frequently produced.

**The Cocci in Skin Diseases.**—Sabouraud, of Paris,[1] said, at the annual meeting of the British Medical Association at Cheltenham, that he considered there were three bacteria of the "coccus" group which were mainly concerned in the production of skin diseases:

1. A streptococcus, which was identical with the streptococcus of Fehleisen, and capable of showing wide variations of virulence. It was the "specific organism" of erysipelas and the allied diseases, and was concerned in the production and establishment of the phenomena of elephantiasis. He thought the specific lesion of the streptococcus in the skin proper was the "phlyctene," or intra-epithelial vesicle or bulla of true impetigo—the impetigo of Tilbury Fox,—the disease which subsequently formed the coin or seal-like crusts seen on the face of children. He also considered that this streptococcus was the origin of certain lesions producing superficial ulceration and crust formation, and that it was also a complicating factor in many inflammatory affections of the skin.

2. The staphylococcus pyogenes aureus of Rosenbach. This organism was concerned in the production of the majority of cutaneous suppurative diseases, in carbuncle and furuncle, in many cases of acne, especially where necrosis of tissue was noticeable, as in acne varioliformis, in forms of suppurative folliculitis, &c. It was also the special cause of that form of impetigo known as the impetigo of Brockhart, in which the pustular lesion commenced as an inflammation in or surrounding a hair-follicle.

3. A coccus growing in greyish-white cultures, which he preferred to designate as the staphylococcus cutis communis. This coccus was found in great numbers on the skin, and must be considered as the cause of the mildly inflammatory affections accompanied by desquamation, such as are typified by pityriasis capitis, seborrhæa corporis, the circinate seborrhœic dermatitis of English writers, and was identical with the coccus described by Unna as the morococcus, and claimed to be the specific organism of nearly all forms of eczema. With this latter hypothesis Sabouraud entirely disagrees.

**Some Observations on the Pathology and Treatment of Corns.**—Under this title Mr. E. Harding Freeland[2] gives some useful information, especially from a surgical standpoint. He says that one of the first changes observed is a proliferation of the cells of the stratum mucosum over a limited area—a change, he thinks, brought about by nervous influence, and the result of a stimulus directly applied from without to the nerve terminals which govern the nutrition of the part affected. As successive layers of cells are added from below more quickly than they are

[1] Reported in the *Brit. J. Dermat.*, 1901, xiii. 348.
[2] *Brit. M. J.*, 1901, n.s., x. 431.

shed on the surface, the superficial cells become heaped up into a mound-like eminence which projects above the normal level of the skin. As the summit of the mound is the oldest, most highly keratinised, and consequently most brittle part, and owing to the fact that its exposed position renders it more liable to attrition, this part of the corn soon becomes detached, breaking off short just below the level of the surrounding tissue, and leaving a shallow, cup-like depression with a roughened surface. The result of this change is, that the chief pressure is shifted from the centre of the corn to the margin of the cup-like depression.

As palliative treatment he recommends Unna's plan. A ring of glycerine jelly is painted round the circumference of the corn so as to form a raised rampart. A piece of salicylic plaster mull is then cut to the size and shape of the central depression, and applied to the surface of the corn. This is then covered with a layer of glycerine jelly, and before it sets a pad of cotton wool is applied to the surface. This process is repeated as often as is necessary, until the horny layer of the corn separates and is cast off. The method of removal in favour with chiropodists is to introduce an instrument at the groove which runs round the margin of the corn, and by making this penetrate towards its central axis with the exercise of a little manual dexterity the horny part of the corn can be easily separated from the parts beneath. As Mr. Freeland says, a corn which has been partially extracted by this method corresponds to breaking the main stem of a shrub short off just below the surface of the ground. The removal of the growth is only partial; the essential part of the corn—the part from which it springs—being left behind.

Mr. Freeland goes on to say that any method of treatment to be curative must secure the removal of the entire corn, together with the underlying bursa, if a bursa is present. He renders the skin insensitive by the application of ethyl chloride, and 5 minims of a 4 per cent. solution of eucaine is injected into the subcutaneous tissue beneath the corn. Under strict antiseptic precautions two hemi-elliptical incisions meeting at their extremities are made through the skin around the circumference of the growth, care being taken that they penetrate well into the subcutaneous tissue. The whole corn is now dissected out, and the edges of the wound brought together in the usual way.

Mr. Freeland thinks that if practitioners of medicine would take a more intelligent interest in the study of these little tumours, the treatment of which legitimately belongs to the domain of surgery, these cases would be gradually lifted from the depths of charlatanism into which they have long since subsided. He says the moral is obvious—both the profession and the public would be the gainers.

The Professor of Pathology at the Army Medical School at Netley has been inoculating patients suffering from furunculosis, sycosis, and acne with a staphylococcus vaccine.

He concludes that much that is of value can be learned upon this subject by a careful consideration of Koch's tuberculin inoculations and by a comparison of these with the inoculations of staphylococcus vaccine. In each case a bacterial vaccine is, for therapeutic purposes, inoculated into patients already the subject of a corresponding infection. In each case, as the result of the inoculation, an acute inflammatory reaction is set up at the seat of the infection. In each case, again as a result of the inflammatory reaction in question, the nidus in which the bacteria are lodged is broken up.

At this point Professor Wright says an all-important difference emerges. In the case where the localised inflammation process is due to a staphylococcus infection the breaking up of the nidus has involved, so far as has hitherto appeared, a destruction of the bacteria which have been affected. In the case of a tubercle infection the destruction of the nidus has, on the other hand, been shown to be compatible with the continued survival of the tubercle bacilli. The probable cause of this difference is thought to be owing to the fact that the tubercle bacillus possesses, as compared with the staphylococcus, an infinitely greater capacity for maintaining its vitality in the interior of the organism. It follows from this that there is much less chance of new localised foci of infection, or, in exceptional cases, of generalisation of infection with a staphylococcus vaccine than with tuberculin inoculation.

In applying an inoculation treatment to the forms of erysipelas referred to in his paper, it would be necessary to keep in view the fact that the streptococcus possesses, as compared with the staphylococcus, a much greater capacity for generalising itself in the organism. Professor Wright mentions details of inoculation treatment in six patients with good results in five, and concludes by suggesting whether or not any therapeutic advantage could be derived from the inoculation of the appropriate bacterial vaccine into patients suffering from chronic "surface-invasions." He says there would be opportunity of applying such inoculations in connection with the treatment of bronchitis, ozœna, gleet, leucorrhœa, and those forms of bacteruria which depend upon a bacterial invasion of the mucous membranes of the genito-urinary tract.

HENRY WALDO.

[1] *Lancet*, 1902, i. 874.

